# Age Prediction of Human Based on DNA Methylation by Blood Tissues

**DOI:** 10.3390/genes12060870

**Published:** 2021-06-06

**Authors:** Jiansheng Zhang, Hongli Fu, Yan Xu

**Affiliations:** Department of Information and Computer Science, University of Science and Technology Beijing, Beijing 100083, China; s20190764@xs.ustb.edu.cn (J.Z.); 18401616742@163.com (H.F.)

**Keywords:** DNA methylation, CpG sites, gradient boosting regression

## Abstract

In recent years, scientists have found a close correlation between DNA methylation and aging in epigenetics. With the in-depth research in the field of DNA methylation, researchers have established a quantitative statistical relationship to predict the individual ages. This work used human blood tissue samples to study the association between age and DNA methylation. We built two predictors based on healthy and disease data, respectively. For the health data, we retrieved a total of 1191 samples from four previous reports. By calculating the Pearson correlation coefficient between age and DNA methylation values, 111 age-related CpG sites were selected. Gradient boosting regression was utilized to build the predictive model and obtained the R^2^ value of 0.86 and MAD of 3.90 years on testing dataset, which were better than other four regression methods as well as Horvath’s results. For the disease data, 354 rheumatoid arthritis samples were retrieved from a previous study. Then, 45 CpG sites were selected to build the predictor and the corresponded MAD and R^2^ were 3.11 years and 0.89 on the testing dataset respectively, which showed the robustness of our predictor. Our results were better than the ones from other four regression methods. Finally, we also analyzed the twenty-four common CpG sites in both healthy and disease datasets which illustrated the functional relevance of the selected CpG sites.

## 1. Introduction

Aging is a natural and irreversible process that occurs throughout a person’s life, and it is influenced by many factors, such as genetic factors, living environment and diseases [1,2]. It is modified and regulated by a variety of molecular modifications occurred in tissues or organs, including chemical modifications and changes in DNA levels such as DNA methylation [3]. In recent years, it is reported that many aging-related performances are formed in the process of a person’s growth through clinical research [4,5]. DNA methylation is catalyzed by a family of DNA methyltransferases (Dnmts) that transfer a methyl group from S-adenyl methionine (SAM) to the fifth carbon of a cytosine residue to form 5mC [6,7]. DNA methylation is one of the earliest and most common modifications for mammalian genomic DNA. It may exist in all higher organisms and play an important regulatory role in gene expression, involving many complex biological processes [5,8]. In 1967, Berdvshev and his team began to explore the relationship between DNA methylation and aging by studying the hunchback carp in the spawning period [9,10]. Subsequently, Vanyushin, Wilson, Bocklandt and other scientists studied with animal and human tissue cells and confirmed that the degree of DNA methylation in different tissues had a certain correlation with age [11,12]. More recently, different models using the degree of DNA methylation have been built for age prediction in various tissues [5,13,14].

In forensic science, individual age has always been an important research indicator. At present, forensic doctors usually use the well-matched models to estimate and predict the age of the individual by measuring bone morphological indicators [15,16,17]. However, sometimes the perpetrators fled after the crime, only leaving sporadic blood, saliva or semen, and the bone markers cannot be found. Thus, it is not feasible to use the above methods to predict age sometimes. Meanwhile, in molecular biology, characteristics such as the degree of DNA damage, mitochondrial mutations and leukocyte telomere length can be used to predict age [18]. Except, in fact, these models are not very effective in predicting ages, and the results are not very satisfactory. Besides, it is not easy to implement on the technical level. Therefore, it is imperative to find another feasible method to predict age. In recent years, with the development of epigenetics, researchers have found that there is a correlation between DNA methylation and aging. With the gradual improvement in DNA methylation research and more in-depth research in this field, the quantitative statistical relationship between DNA methylation and different ages was well established according to the change of DNA methylation with age [19,20].

Based on previous studies, Horvath et al. used the degree of DNA methylation in various human tissues to predict the actual age of an individual [21]. Horvath et al. selected 7844 samples from different tissues and cell types, and performed an intensive analysis on relevant experiments and information data to study the correlation between the degree of DNA methylation and age. Finally, they selected 353 CpG sites common in several different tissues and identified that DNA methylation levels of these 353 CpG sites were predictive for estimating human age. Specifically, they used this set of sites to successfully construct an age predictor across different tissue types, with a mean absolute deviation (MAD) value of 3.6 years [13,21,22]. Following Horvath’s seminal study, a large number of scientists began to engage in and contribute to this field. For instance, in 2014, Dr. Yi and his team used blood samples to predict age with a multiple linear regression, and the MAD was about 4 years [23]. Zbiec-Piekarska et al. built an age predictor by using human blood CpG sites with a multiple linear regression model in 2015 [24,25,26,27]. Different from their strategies where linear regression models were used, we adopted a nonlinear regression model called gradient boosting regression to build the age predictor. Through comparing R^2^, MAD, MSE and RMSE (four performance indicators for regression) on training sets and testing sets, our non-linear age predictor performed better than linear regression models.

## 2. Materials and Methods

### 2.1. Data Collection and Processing

We downloaded four datasets from the National Center for Biotechnology Information (NCBI) Gene Expression Omnibus (GEO). All of these datasets were selected from Illumina Human Methylation 450 BeadChip. Here are some details about healthy and disease dataset (Table 1). The healthy datasets contain a total of 1191 healthy individuals and the disease dataset has a total of 354 rheumatoid arthritis patients.

β values of DNA methylation were used in all experiments. For each CpG site the β value ranged between 0 and 1 indicates the ratio of methylation. Where 1 represents complete methylation, and 0 represents complete demethylation. The data processing was following: (1) extract relevant information (including age and the β value) from the original datasets downloaded from GEO; (2) merge four datasets and impute in the missing value. For each CpG site if there were ≥30 samples missing, we removed it. Otherwise, we imputed the missing values with the average of that CpG site.

### 2.2. Selection of Age-Related CpG Sites for Healthy Blood and Rheumatoid Arthritis Disease Dataset

To illustrate the performance of different models, we randomly divided the benchmark dataset into training and testing in a ratio of 7:3. CpG sites were selected as following: (1) calculate Pearson correlations between human age and DNA methylation value of each CpG site in the training; (2) choose the CpG sites whose Pearson correlation was more than 0.6 or less than −0.6. According to Pearson correlation analysis, 111 highly age-related CpG sites [32,33] were selected (Supplementary S1). The disease data were dealt with the same scheme as healthy samples. 45 CpG sites were selected with Pearson correlation absolute values greater than 0.6 (Supplementary S2).

### 2.3. Operation Algorithm

Based on the idea of boosting algorithm, Friedman came up with the gradient boosting regression (GBR) algorithm [34]. Nowadays, GBR is widely applied in the field of biology. It is precisely because GBR can effectively process data with noise and support different loss function. In addition to this, GBR also provides better accuracy for predicting data, especially in terms of non-linear data. GBR is a non-parametric supervised machine learning algorithm, and it approximates the unknown functional mapping from input explanatory variables to corresponding output variables [35]. The key of GBR is to use the negative gradient of the loss function in the current model [36]. Besides, we chose the minimum absolute deviation as the loss function, L(y,f(X)).

(1) $$L\left(y,f\left(X\right)\right)=\frac{1}{2}\left|y-f\left(X\right)\right|$$

where X is the input vectors, y is the output vector, and the regression function is;

(2) $$\sum_{t=1}^{T}f_{t}\left(X\right)=\sum_{t=1}^{T}\beta_{t}h\left(X;\alpha_{t}\right)$$

where T is the number of basic functions, t is the ordinal number (t from 1 to T), $\beta_{t}$ is the expansion coefficient, α represents the node branch variable and $h\left(X;\alpha_{t}\right)$ is the basis function with fewer parameters and simple. We utilized the sklearn package in python and the parameters are as following:

learning_rate = 0.03, n_estimators = 400, subsample = 0.6, min_samples_split = 2, max_depth = 4, alpha = 0.6, verbose = 0.

### 2.4. Statistical Measurement

In machine learning, performance indicators are the key to measure the quality of a predictor. Performance indicators reflect the task requirements. When comparing the capabilities of different predictors, different performance indicators often lead to different evaluation results. What kind of model is good, not only depends on algorithms and data but also task requirements. In this work, we used the common following performance indicators for regression [20,25]:

(3) $$\left\{ \begin{array}{c} R^{2}=1-\frac{\sum_{i=1}^{m}{\left(y_{i}-f\left(x_{i}\right)\right)}^{2}}{\sum_{i=1}^{m}{\left(y_{i}-\bar{y}\right)}^{2}}\\ \mathrm{MAD}=\frac{\sum_{i=1}^{m}\left|y_{i}-\bar{y}\right|}{m}\\ \mathrm{MSE}=\frac{\sum_{i=1}^{m}{\left(y_{i}-\bar{y}\right)}^{2}}{m}\\ \mathrm{RMSE}=\sqrt{\frac{\sum_{i=1}^{m}{\left(y_{i}-\bar{y}\right)}^{2}}{m}} \end{array}\right.$$

where m represents the number of samples, $y_{i}$ is the actual age and $\bar{y}$ is the predicted age. The MAD is the mean absolute deviation between the predicted age and the actual age, MSE is mean square error, RMSE is root mean square error and R^2^ is correlation coefficient.

## 3. Results

### 3.1. Results of Healthy Blood Tissues

**Figure 1:**
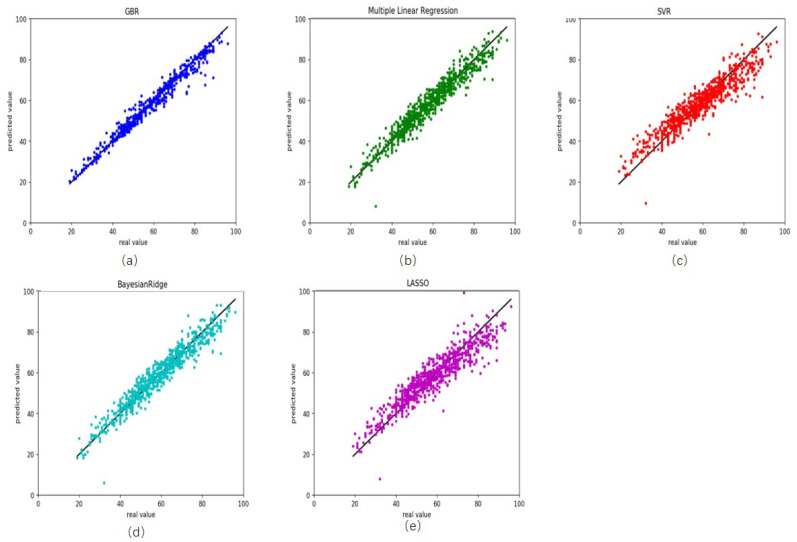
Comparison results between the actual age and predicted age by five different models on the training health data. (**a**) gradient boosting regression, (**b**) multiple linear regression, (**c**) support vector regression, (**d**) Bayesian ridge regression and (**e**) lasso regression.

To illustrate the performance of gradient boosting regression, we compare it with other four common regression models multiple linear regression [37,38], support vector regression [39], Bayesian ridge regression [40] and lasso regression [41]. On the training, R^2^ was 0.97 for gradient boosting regression, with root mean square error (RMSE) and MAD being 2.46 and 1.40 years, respectively (Figure 1a and Table 2). The RMSE and MAD were 3.83 and 2.91 years for multiple linear regression (Figure 1b), 5.54 and 4.20 years for support vector regression (Figure 1c), 3.88 and 2.94 years for Bayesian ridge regression (Figure 1d), 5.57 and 4.19 years for lasso regression (Figure 1e).

**Figure 2:**
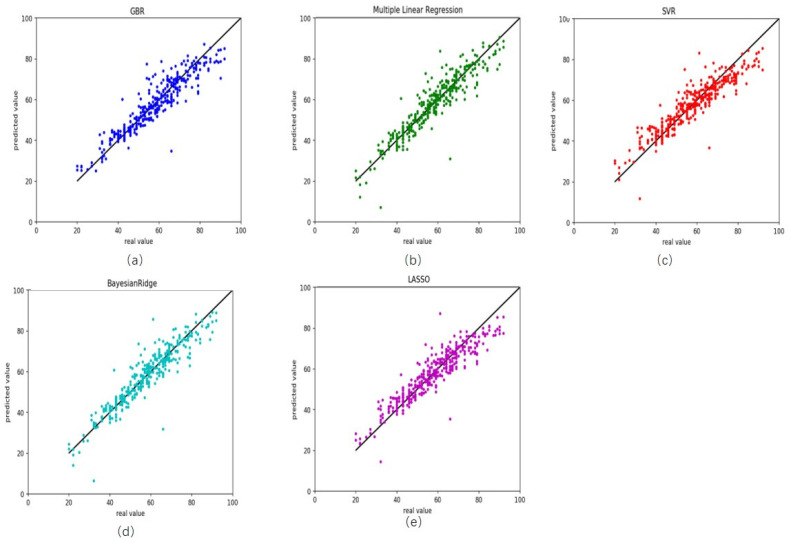
Comparison results between the actual age and predicted age by five different models on the testing healthy data. (**a**) gradient boosting regression, (**b**) multiple linear regression, (**c**) support vector regression, (**d**) Bayesian ridge regression and (**e**) lasso regression.

On the testing dataset, these results were similar to those in training (Table 2). R^2^ was 0.86 for gradient boosting regression, with RMSE and MAD being 5.54 and 3.90 years, respectively (Figure 2a). The RMSE and MAD were 5.49 and 2.92 years for multiple linear regression (Figure 2b), 5.94 and 4.44 years for support vector regression (Figure 2c), 5.33 and 3.67 years for Bayesian ridge regression (Figure 2d) and 5.82 and 4.34 years for lasso regression (Figure 2e). In this work, we also compared our results with that of Horvath [21] (hereinafter referred to as Horvath’s), the current state-of-the-art. Horvath’s MAD was 4.9441 and RMSE 6.4119. Our results were better than those ones which showed the performance and robustness of our predictor on healthy blood tissues.

### 3.2. Results of Rheumatoid Arthritis Disease

**Figure 3:**
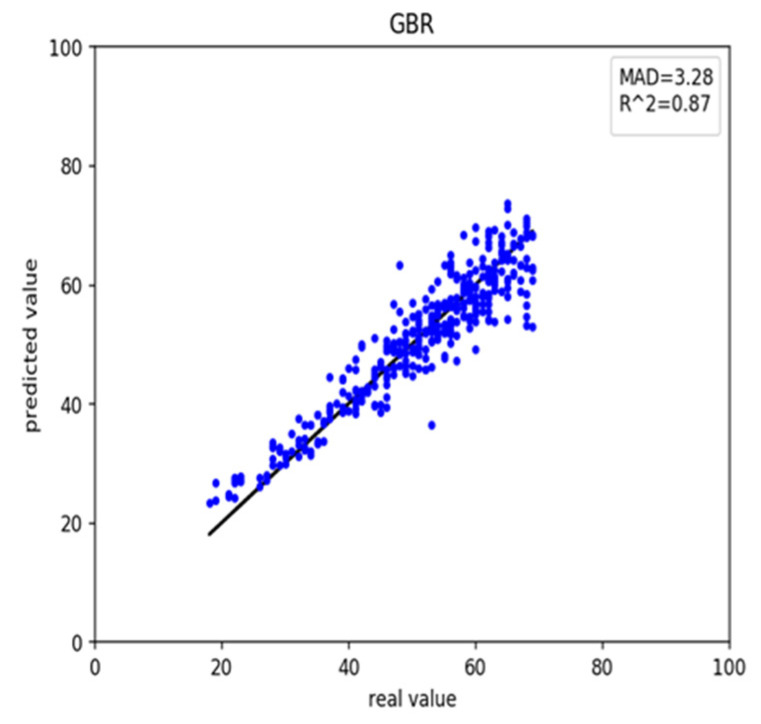
The scatter plot of the rheumatoid arthritis dataset with the healthy predictor.

**Figure 4:**
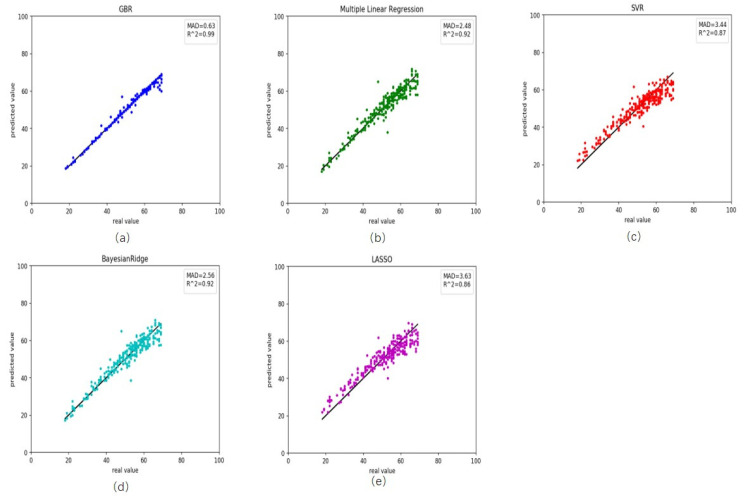
Comparison results between the actual age and predicted age by five different models on the training rheumatoid arthritis data. (**a**) gradient boosting regression, (**b**) multiple linear regression, (**c**) support vector regression, (**d**) Bayesian ridge regression and (**e**) lasso regression.

**Figure 5:**
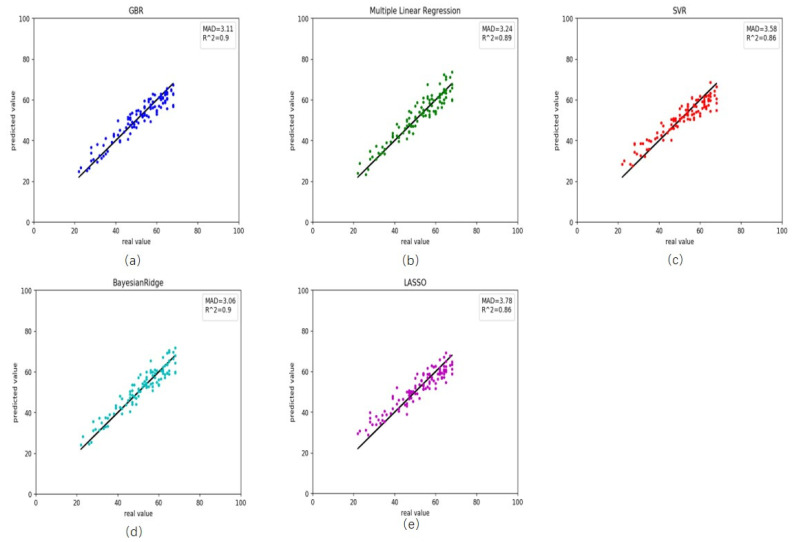
Comparison results between the actual age and predicted age by five different models on the testing rheumatoid arthritis data. (**a**) gradient boosting regression, (**b**) multiple linear regression, (**c**) support vector regression, (**d**) Bayesian ridge regression and (**e**) lasso regression.

We also retrieved rheumatoid arthritis disease data from GEO. First, we used the healthy predictor to predict the rheumatoid arthritis data. The RMSE and MAD were 18.69 and 3.28 years, respectively (Table 3). These results and scatter plot (Figure 3) which samples were near the central straight line could be accepted. However, rheumatoid arthritis data could have its characters and a specific impact on DNA methylation. As a result, we recalculated the Pearson correlation and select 45 CpG sites, then retrained the GBR. On the training, the RMSE and MAD were 1.46 and 0.63 years for gradient boosting regression (Figure 4a), 3.34 and 2.48 years for multiple linear regression (Figure 4b), 4.40 and 3.44 years for support vector regression (Figure 4c), 3.42 and 2.56 years for Bayesian ridge regression (Figure 4d) and 4.56 and 3.63 years for lasso regression (Figure 4e). These results improved greatly (Table 4). Meanwhile, on the testing the RMSE and MAD were 3.90 and 3.11 years for gradient boosting regression (Figure 5a), 4.06 and 3.24 years for multiple linear regression (Figure 5b), 4.47 and 3.58 years for support vector regression (Figure 5c), 3.82 and 3.06 years for Bayesian ridge regression (Figure 5d) and 4.57 and 3.78 years for lasso regression (Figure 5e). The RMSE and MAD for gradient boosting regression improved 14.79 and 0.17, respectively. The performance of the retrained predictor was better than the former healthy ones on rheumatoid arthritis data.

### 3.3. Impact of Disease on Age Prediction

As we all know, some genes are linked to age-related diseases, such as cancer and Alzheimer’s disease. DNA methylation is not regular in these diseases. Dr. Horvath’s experiment showed that the predicted age of cancer patients had poor correlation with the actual age [21]. Park and his team found that the correlation between the degree of methylation and age of three CpG sites in patients with acute myeloid leukemia disappeared [24,42]. There were also studies showing that Alzheimer’s disease had a certain correlation with some age-related DNA methylation [43,44]. In this work, the impact of disease on age prediction was mainly reflected in the repeated twenty-four CpG sites (Table 5). The twenty-four common CpG sites between healthy and disease dataset indicated that arthritis disease affected DNA methylation and had a correlation with age. However, other twenty-one new sites have obtained this correlation.

### 3.4. Analysis of Selected Twenty-Four CpG Sites

**Figure 6:**
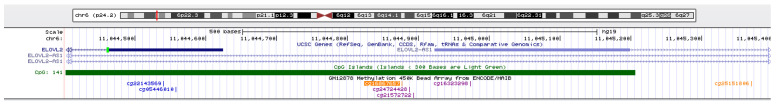
UCSC genome browser view of the genomic location of the CpG cg16867657.

A total of twenty-four CpG sites in the rheumatoid arthritis disease were identical to the healthy dataset which may be the reason why disease dataset can also be applied to healthy predictor and obtained accepted performance. In order to find out the effect of these twenty-four CpG sites on age, we performed biological analysis on these sites and visualized them on UCSC genome browser (https://genome.ucsc.edu/, accessed on 20 October 2020). For example, it can be seen from the Figure 6 that cg16867657 was located in Human Gene ELOVL2. Besides, from the Table 5, we can see that several CpG sites mainly locate in Human Gene ELOVL2 and FHL2, which are considered as age-related genes, and play important roles in the process of human aging [42,45,46,47]. In fact, we observed that all these 24 CpGs were basically located on the age-related genes, implied their functional relevance with age.

## 4. Discussion

At present, age prediction becomes more and more popular in the field of DNA methylation. In the last decade, many studies have been conducted in the field, and there were several age predictors. In 2009, based on human blood sample data, Bekaert et al. established a quadratic regression model of age predictor, and accuracy of the predictor reached the high level at that time. Interestingly, they found the accuracy decreased with age increasing [48]. From 2013 to 2015, Horvath, Yi and Zbiec-Piekarska built linear models to predict age [21,23,24]. The advantage of linear models was that they were fast and easy to use. In 2017, Alisch et al. brought in non-linear models and built non-linear age predictor. Since they only used children dataset (3–17 years old), their model could not be applied to all age groups. They also found that the DNA methylation did not change at a constant rate with age in life [49]. Here, we intend to establish an age predictor that uses a nonlinear model and is suitable for all age groups.

In this work, we selected 111 CpG sites through calculating Pearson correlation in the healthy datasets. The predictor based on gradient boosting regression has better performance than other four models. In the disease dataset, we used a dataset of rheumatoid arthritis patients with a total of 354 samples. There were twenty-four common CpG sites between healthy and disease dataset, indicating that age-related diseases may have some effects on DNA methylation. The performance of new predictor improved greatly with disease CpG sites which showed rheumatoid disease having its certain correlation with age-related DNA methylation.

Of course, there were still some limitations in this study. First, the impact of gender on DNA methylation and age was not considered. In the past, scientists held two very different perspectives on gender research. Zaghlool SB showed that age-related methylation levels may differ in gender performance [48]. However, in Bram’s study [24], between men and women, age-related methylation levels seemed to be similar. Secondly, we did not consider the effects of environmental factors. Jenkins et al. studied DNA methylation in male sperm, found that long-term smoking and harsh environments (such as severe cold) accelerate the aging of gametes, making the predicted age often higher than the actual age [13,47,49]. Thirdly, we only used blood tissue, did not use data from other organs, such as skin, lungs and so on. Song et al. found each tissue had a different methylation pattern [21,50], implied that tissue-specific age predictors might achieve better performance than the multiple-tissue one. Finally, some age-related diseases and cancers were shown to accelerate or slow down the degree of DNA methylation [51]. Our disease dataset only contained one disease, leaving it being less explored whether other diseases affect age. In future, we will continue the work from the above aspects.

## 5. Conclusions

Age prediction based on DNA methylation was rapidly evolving in the field of epigenetics. In this work, we collected four healthy datasets and selected 111 highly age-associated CpG sites by calculating the Pearson correlation between age and DNA methylation value of each CpG site. Through comparing with other four regression algorithms, our proposed GBR was optimal which achieved R^2^ value of 0.97 and MAD of 1.40 years on training datasets, and R^2^ of 0.86 and MAD of 3.90 years on testing datasets, respectively. For the rheumatoid arthritis disease dataset, we identified 45 CpG sites showing highest Pearson correlations. The MAD and R^2^ were 0.63 years and 0.98 with GBR on the training dataset, and 3.11 years and 0.89 on the testing dataset. In addition, the deep analysis of twenty-four common CpG sites for both healthy and rheumatoid arthritis disease datasets illustrated the importance of the selected CpG sites.

## Figures and Tables

**Table 1:** Four healthy and one disease DNA methylation datasets.

Series	DNA Origin	Platform	Author and Publication Year	Disease	Number
GSE40279	Blood	450k	Zhang K [28] (2012)	--	656
GSE42861	Blood	450k	Liu Y [29] (2013)	--	335
GSE65638	Blood	450k	Xu C [30] (2015)	--	16
GSE69270	Blood	450k	Kananen L [31] (2016)	--	184
GSE42861	Blood	450k	Liu Y [29] (2013)	Rheumatoid arthritis	354

**Table 2:** Comparison results of our predictor with other four regression models and Horvath’s model on healthy datasets.

	R^2^	MAD	MSE	RMSE
Training				
Multiple Linear Regression	0.9363	2.9150	14.647	3.8271
Support Vector Regression	0.8667	4.1965	30.636	5.5350
Bayesian Ridge Regression	0.9345	2.9376	15.064	3.8813
Lasso Regression	0.8652	4.1925	30.982	5.5661
Gradient Boosting Regression	0.9737	1.4034	6.0335	2.4563
Testing				
Multiple Linear Regression	0.8649	3.8228	30.1042	5.4867
Support Vector Regression	0.8417	4.4448	35.2690	5.9387
Bayesian Ridge Regression	0.8727	3.6679	28.3670	5.3260
Lasso Regression	0.8478	4.3360	33.9035	5.8226
Gradient Boosting Regression	0.8625	3.8988	30.6367	5.5350
Horvath’s model	0.8110	4.9441	41.1128	6.4119

**Table 3:** Performance of the healthy predictor on rheumatoid arthritis dataset.

R^2^	MAD	MSE	RMSE
0.870958	3.284863	18.691550	4.323373

**Table 4:** Comparison results of our predictor with other four common regression models on rheumatoid arthritis dataset.

	R^2^	MAD	MSE	RMSE
Training				
Multiple Linear Regression	0.922834	2.477032	11.16546	3.341476
Support Vector Regression	0.866253	3.439445	19.35249	4.399147
Bayesian Ridge Regression	0.919139	2.564907	11.70018	3.420553
Lasso Regression	0.856411	3.625878	20.77659	4.558135
Gradient Boosting Regression	0.985262	0.625448	2.132504	1.460310
Testing				
Multiple Linear Regression	0.886814	3.242406	16.46903	4.058205
Support Vector Regression	0.862663	3.582393	19.98303	4.470239
Bayesian Ridge Regression	0.899453	3.064368	14.62997	3.824914
Lasso Regression	0.856548	3.780038	20.87289	4.568686
Gradient Boosting Regression	0.895673	3.114274	15.18006	3.896159

**Table 5:** Information about the twenty-four common CpG sites for healthy and rheumatoid arthritis datasets.

CpG Sites	Pearson Correlation Coefficient in Healthy Datasets	Pearson Correlation Coefficient in Disease Datasets	Physical Position in GRCh37/hg19 (Chromosome: Position)	Gene Names
cg16867657	0.8715	0.8240	chr6:11044877	*ELOVL2*
cg22454769	0.7892	0.8107	chr2:106015768	*FHL2*
cg19283806	−0.7646	−0.7112	chr18:66389420	*CCDC102B*
cg04875128	0.7412	0.6803	chr15:31775896	*OTUD7A*
cg10501210	−0.7381	−0.7302	chr1:207997020	-
cg24079702	0.7328	0.6829	chr2:106015772	*FHL2*
cg06639320	0.7265	0.8027	chr2:106015740	*FHL2*
cg08097417	0.7019	0.6814	chr7:130419134	*KLF14*
cg07082267	−0.6933	−0.6650	chr16:85429036	-
cg24724428	0.6788	0.6607	chr6:11044888	*ELOVL2*
cg09809672	−0.6723	−0.6005	chr1:236557683	-
cg11649376	−0.6667	−0.6361	chr12:81473234	*ACSS3*
cg23078123	−0.6587	−0.6089	chr1:68577796	*GNG12*
cg08262002	−0.6525	−0.6530	chr4:16575323	*LDB2*
cg21572722	0.6503	0.8270	chr6:11044894	*ELOVL2*
cg18933331	−0.6463	−0.6085	chr1:110186419	-
cg06784991	0.6427	0.6287	chr1:53308769	*ZYG11A*
cg22736354	0.6370	0.6769	chr6:18122719	*NHLRC1*
cg01528542	−0.6250	−0.6350	chr12:81468232	-
cg23500537	0.6093	0.7347	chr5:140419820	-
cg06819923	−0.6087	−0.6300	chr16:21214509	*ZP2*
cg17110586	0.6035	0.6934	chr19:36454623	-
cg00481951	0.6031	0.6107	chr3:187387651	*SST*
cg03473532	−0.6012	−0.6310	chr7:131008744	*MKLN1*

## Data Availability

Not applicable.

## Supplementary Materials

The following are available online at https://www.mdpi.com/article/10.3390/genes12060870/s1, Supplementary S1: 111 selected CpG sites on the healthy dataset, Supplementary S2: 45 selected CpG sites on the disease dataset.
