# Supplementary material for: Age Prediction of Human Based on DNA Methylation by Blood Tissues
Source: Genes (Basel). 2021 Jun 6;12(6):870. doi: 10.3390/genes12060870 (PMC8228382; doi:10.3390/genes12060870)
Supplement: Supplementary file 1 [file genes-1219900-supplementary.pdf › genes-1219900-supplementary.pdf]

Response to Reviewer 2 Comments

The manuscript by Zhang et al. entitled "Age prediction of human based on DNA methylation by blood tissues" is a report on age-predicting genomic DNA methylation sites selected by 1191 healthy and disease-affected samples using gradient boosting regression. The study has reported 24 common CpG sites between both datasets. These findings will be to interest of the readers of Gene, especially DNA methylation researchers in the fields of human geneticists, cancer research, and forensic science. I have, however, major and minor comments to be addressed before publication. I hope these comments will be helpful.

Major comments:

Point 1. In Table 5, Supplementary S1 and S2, Illumina's CpG loci IDs are not very familiar to the readers. Add information of chromosome, coordinate (physical position), as well as reference version (GRCh36, hg18, or newer) to these tables. Newer reference versions are preferable.

Response: Thanks for your suggestion. We have added some information about the CpG sites in Table 5, which includes Pearson correlation coefficient both in healthy datasets and disease datasets, physical position, Gene name, and reference versions.

Table 5. Information about the twenty-four same CpG sites for healthy and disease datasets.

CpG site	Pearson correlation coefficient in healthy datasets	Pearson correlation coefficient in disease datasets	Physical position (chr : position)	Gene names	Reference versions
cg16867657	0.8715	0.8240	chr6:11044877	ELOVL2	GRCh37/hg19
cg22454769	0.7892	0.8107	chr2:106015768	FHL2	GRCh37/hg19
cg19283806	-0.7646	-0.7112	chr18:66389420	CCDC102B	GRCh37/hg19
cg04875128	0.7412	0.6803	chr15:31775896	OTUD7A	GRCh37/hg19
cg10501210	-0.7381	-0.7302	chr1:207997020	-	GRCh37/hg19
cg24079702	0.7328	0.6829	chr2:106015772	FHL2	GRCh37/hg19
cg06639320	0.7265	0.8027	chr2:106015740	FHL2	GRCh37/hg19
cg08097417	0.7019	0.6814	chr7:130419134	KLF14	GRCh37/hg19
cg07082267	-0.6933	-0.6650	chr16:85429036	-	GRCh37/hg19
cg24724428	0.6788	0.6607	chr6:11044888	ELOVL2	GRCh37/hg19
cg09809672	-0.6723	-0.6005	chr1:236557683	-	GRCh37/hg19
cg11649376	-0.6667	-0.6361	chr12:81473234	ACSS3	GRCh37/hg19

cg23078123	-0.6587	-0.6089	chr1:68577796	GNG12	GRCh37/hg19
cg08262002	-0.6525	-0.6530	chr4:16575323	LDB2	GRCh37/hg19
cg21572722	0.6503	0.8270	chr6:11044894	ELOVL2	GRCh37/hg19
cg18933331	-0.6463	-0.6085	chr1:110186419	-	GRCh37/hg19
cg06784991	0.6427	0.6287	chr1:53308769	ZYG11A	GRCh37/hg19
cg22736354	0.6370	0.6769	chr6:18122719	NHLRC1	GRCh37/hg19
cg01528542	-0.6250	-0.6350	chr12:81468232	-	GRCh37/hg19
cg23500537	0.6093	0.7347	chr5:140419820	-	GRCh37/hg19
cg06819923	-0.6087	-0.6300	chr16:21214509	ZP2	GRCh37/hg19
cg17110586	0.6035	0.6934	chr19:36454623	-	GRCh37/hg19
cg00481951	0.6031	0.6107	chr3:187387651	SST	GRCh37/hg19
cg03473532	-0.6012	-0.6310	chr7:131008744	MKLN1	GRCh37/hg19

Point 2. In line 169-170, I cannot find gene annotation of each 23 CpG site. In addition to genomic positions, add information of nearly positioned genes (for example, within 1kb upstream and downstream around a CpG site) to Table 5. If some CpG sites are located near age-related genes, indicate those with proper citation. Interpretation of these results should be described in the Discussion section. If not all of the 23 CpG site are located near age-related genes, the sentence "... have a strong correlation with age" sounds too strong.

Response: In line 169, after "…cg16867657 is located in Human Gene ELOVL2", we have added "Besides, from the Table 5, we can see that the twenty-four CpG sites mainly locate in Human Gene ELOVL2 and FHL2, which are considered as age-related genes". In this part, we also selected some relevant articles and cited them (Ref 40, Ref 43-45) in the references.

Minor comments:

Point 1. In line 11-13 in the Abstract section, after "...we retrieved a total of 1191 samples.", add words "of five previous reports from a public database".

Response: Thank you for your comments. We have revised lines 11-13 in the Abstract section as "we retrieved a total of 1191 samples of five previous reports from a public database".
